# 7.M. Round table: Preparedness & response for emergency situations: joined forces of H2020projects in the PREPAREcluster

**DOI:** 10.1093/eurpub/ckac129.449

**Published:** 2022-10-25

**Authors:** Organised by: PHIRI, Sciensano (Belgium), EUPHA-PHMR, Chair persons: Svetla Tsolova (Sweden), Petronille Bogaert (EUPHA-PHMR)

## Abstract

In recent years, there have been increasing instances of cross-border crises, including climate change, terrorism, international trade disputes and global health threats. These emergency situations require large-scale planning for preparedness and response in order for countries to be able to cope with unforeseen challenges. Especially the COVID-19 crisis had a huge impact on European countries and the daily lives of its citizens. The pandemic has proven to be more than a health crisis; it is a human, economic and social crisis, impacting people, societies and economies at their core. The European Commission has recently funded many projects (in the Horizon 2020 program, H2020) to work on different aspects of crisis management, many with a focus on managing pandemics. While each project has distinct aims and challenges, they all work towards a common goal. It is for this reason that thirteen EU-funded H2020 projects, with a combined funding of €72 million, have united to form the PREparedness and resPonse for emergency situAtions in euRopE (PREPARE) cluster. Each of the thirteen projects is tackling challenges specifically looking at the preparedness and response phases of crisis management and working together they aim to achieve stronger results and greater impact for their cause. Together CO-VERSATILE, COVID-X, COVINFORM, EUR3KA, LINKS, NO FEAR, PANDEM-2, PathoCERT, PERISCOPE, PHIRI, RISKPACC, STAMINA and STRATEGY will explore synergies, research opportunities and deliver joint activities to maximise impact. Through mutual support, the cluster will strengthen the response to the ongoing crisis and the aim to be better prepared for future health crises. In a round table discussion, a selection of these H2020 will briefly present their contribution to crisis preparedness and resilience of European countries (25 min). These brief presentations will be followed by a round table discussion (35 min), touching upon topics such as common findings (building trust, health inequalities, training and capacity building, addressing stakeholder diversity); and the dissemination and exploitation of the results to the general public, to researchers, to (public) health professionals and policy makers, Finally, as many of these projects end in 2022/2023, what are the next steps or threats, what should be the highest priority for future Horizon Europe projects? The audience will be able to provide their view on the different topics through an interactive voting poll during the session. Throughout the session, the exchange of knowledge, experiences and opinions with the audience will be facilitated by the chairs.

**Key messages:**

• The actionable outcomes of the major Horizon 2020 projects provide key-input for political decision-making in preparedness and response scenarios.

• The PREPARE cluster builds a sustainable structure for large-scale planning for preparedness and response for countries to be able to cope with unforeseen challenges.

**Speakers/Panellists:**

**Claudia Habl**

Gesundheit Österreich GmbH, Austrian National Public Health Institute, Vienna, Austria

**Brigita Kairiene**

National Public Health Centre, Ministry of Health, Vilnius, Lithuania

**Claudia Houareau**

Robert Koch Institute, Berlin, Germany

**Jil Molenaar**

University of Antwerp, Antwerp, Belgium

**Claim Rafalowski**

Magen David Adom, Or Yehuda, Israel

